# Cognitive fatigue from schema rigidity and entropy externalization: a free energy principle perspective

**DOI:** 10.3389/fpsyg.2025.1556597

**Published:** 2026-01-26

**Authors:** Souhir Ezzedini

**Affiliations:** Laboratory Paragraphe, Université Paris 8, Saint-Denis, France

**Keywords:** brain-environment interaction, cognitive fatigue, cognitive inertia, cognitive schema rigidity, entropy

## Abstract

Cognitive fatigue is typically described as a psychobiological state that arises from intensive and/or prolonged cognitive performance and is experienced as a subjective feeling of exhaustion and lack of energy. It has been predominantly explained through models focused on internal resource depletion or diminished attention. Yet, such perspectives overlook the systemic and dynamic interplay between the brain and the environment. This article introduces a novel theoretical framework grounded in Friston's Free Energy Principle and informed by thermodynamic intuitions about open systems, conceptualizing cognitive fatigue as an outcome of the interaction between cognitive schema rigidity, increasing entropy, and imbalances between internal processes and external environmental demands. Cognitive schemas, which serve as stabilizing mental structures, play a key role in reducing uncertainty, maintaining cognitive stability, and supporting coherent perception–action. When their rigidity—rooted in cognitive inertia—limits their capacity to adapt to environmental changes, it hinders the brain's ability to externalize entropy. This results in an accumulation of unresolved internal disorder and heightened cognitive load, progressively exacerbating fatigue. This theoretical framework emphasizes the importance of brain-environment interactions in mitigating entropy accumulation and highlights the need for greater focus on schema flexibility to enhance cognitive adaptability. By integrating systemic and dynamic perspectives, this framework provides new insights into the mechanisms of cognitive fatigue and its implications for performance in demanding contexts.

## 1 Introduction

Cognitive fatigue is “a psychobiological state induced by prolonged and/or intense cognitive task performance, characterized by a subjective feeling of exhaustion and lack of energy (Boksem and Tops, 2008; Rozand and Lepers, 2017).” Traditional explanations focus primarily on internal processes, such as diminished attention or self-control (Inzlicht et al., 2014; Hockey, 2011) or the ego depletion theory (Baumeister et al., 1998, 2018). A key limitation is that they neglect the complex interplay between the brain and the environment. External factors such as environmental uncertainty can profoundly influence cognitive stability, making it essential to consider a broader, systemic perspective.

Recent research highlights the dynamic interplay between internal cognitive rhythms and external environmental constraints in shaping behavioral and attentional processes (Nobre and van Ede, 2023). This interaction is crucial for the brain's capacity to adapt to changing demands and maintain cognitive stability. Attention plays a central role in this process, functioning as a mechanism to evaluate the precision or uncertainty—conceptualized as entropy within the system—of sensory inputs and modulate information processing accordingly (Feldman and Friston, 2010; Friston, 2013).

According to Friston's predictive coding theory, the brain operates as an inference machine, continually updating its internal models to minimize prediction errors. These internal models—representations of the external world—enable the brain to anticipate sensory inputs and plan actions. Through predictive loops, the brain integrates perception and action, adjusting movements in real-time and preparing for future events. Higher-level generative models guide these predictions, processing information across different temporal and spatial scales to optimize responses to environmental demands (Friston, 2010; Pezzulo et al., 2021).

However, this process is not without limits. Cognitive schemas, which serve as stable mental templates for structuring knowledge and guiding attention, play a pivotal role in maintaining coherence during these interactions (Wei, 2023; Neisser, 1976). These schemas enable the brain to sustain reduced levels of entropy by efficiently organizing information and minimizing uncertainty. To maintain internal order, living systems—including the brain—must actively externalize entropy by offloading disorder to the surrounding environment (Rabeyron, 2021). Building on classical thermodynamics (Carnot, 1824) and on Schrödinger's account of living systems, maintaining internal order requires continual exchange with the surroundings, with entropy ultimately dissipated into the environment (Schrödinger, 1944).

Wei (2023) hypothesizes that cognitive schemas act as stabilizing structures that resist disorder by organizing and structuring information. This hypothesis draws directly from the thermodynamic principle that maintaining internal order in open systems requires continuous interactions with the environment to manage entropy. Wei suggests that this resistance to disorder can be conceptualized as cognitive inertia, where schemas inherently resist change unless influenced by significant external forces. When these schemas become excessively rigid due to high inertia, they lose their adaptive flexibility, making it difficult to externalize entropy—an essential process by which internal disorder is offloaded to the environment to sustain systemic stability. This inertia driven rigidity results in the accumulation of internal entropy, reflecting a mismatch between the brain's internal models and external environmental demands.

By integrating principles from the second law of thermodynamics (Carnot, 1824) and Friston's Free Energy Principle (Friston, 2010, 2013), I propose that cognitive schemas function as stabilizing mental structures that counteract entropy by minimizing prediction errors. These schemas play a crucial role in maintaining cognitive stability by reducing uncertainty and facilitating efficient information processing. This stability can come at a cost. Under prolonged or complex demands, cognitive schemas can become excessively rigid, limiting their adaptability and capacity to align with environmental changes. This rigidity disrupts the brain's ability to externalize entropy, resulting in an accumulation of internal disorder and contributing to increasing cognitive load.

This article explores cognitive fatigue as a phenomenon emerging from the interaction between cognitive schema rigidity, increasing entropy, and imbalances between internal processes and external environmental demands. By incorporating structural and dynamic elements, this framework offers a novel perspective that goes beyond traditional models, emphasizing the systemic and interactive nature of cognitive processes.

Specifically, I argue that cognitive fatigue arises not only from the brain's effort to sustain performance but also from its difficulty in reconfiguring schemas to adapt to changing demands. This perspective highlights the critical role of schema flexibility and the brain's capacity to dynamically engage with its environment. By integrating systemic and environmental dynamics, this approach provides a more comprehensive understanding of cognitive fatigue.

**Figure 1:**
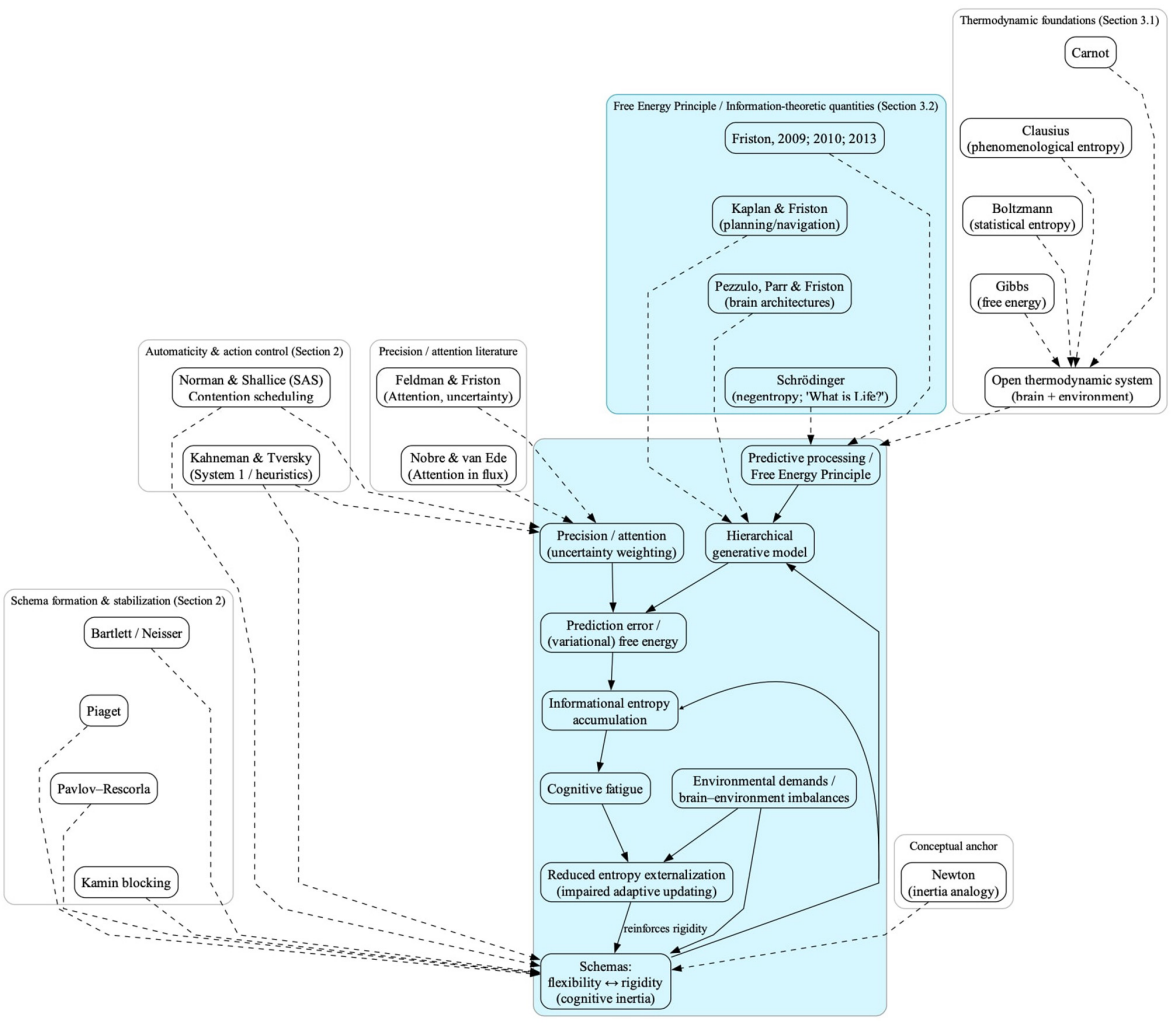
Conceptual map of the *schema-rigidity*/*entropy-regulation* model and its theoretical interfaces. The backbone (solid arrows) depicts the core mechanism. Within a *predictive processing/Free Energy Principle perspective*, the brain relies on a *hierarchical generative model* and weights *uncertainty through precision/attention*. *Prediction errors/(variational) free energy* normally drive internal model updating (the model-updating loop). When *schemas* become rigid (*cognitive inertia*), they constrain updating, promoting sustained prediction errors, rising *informational entropy*, and ultimately *cognitive fatigue*, which can in turn increase reliance on *rigid schemas* (self-reinforcing loop). Peripheral blocks (dashed arrows) form an interface layer around a Fristonian backbone, grounded in the “Free Energy Principle/Information-theoretic quantities” block (Section 3.2). This interface layer includes Section 2—schema formation and stabilization (Bartlett/Neisser, Piaget, Pavlov–Rescorla, Kamin) and automaticity and action control (Norman & Shallice; Kahneman & Tversky)—as well as precision/attention as uncertainty weighting (Feldman & Friston; Nobre & van Ede), thermodynamic foundations converging on an open thermodynamic system (brain + environment; Carnot, Clausius, Boltzmann, Gibbs; Section 3.1), and Newton as a conceptual anchor for the inertia analogy.

In this paper, I aim to build on the theoretical foundations outlined above to develop a comprehensive framework for understanding cognitive fatigue grounded in predictive processing and Friston's Free Energy Principle, while noting where traditional accounts remain underspecified with respect to brain–environment dynamics. I propose a novel perspective that links cognitive schema rigidity to entropy regulation (Figure 1).

First, I introduce cognitive schemas as stabilizing mechanisms that support coherent prediction, and I define cognitive inertia as the tendency of entrenched schemas to resist change, thereby limiting internal model updating and the minimization of prediction error under changing environmental demands. I then explain entropy as informational uncertainty and integrate thermodynamic intuitions about open systems with variational free-energy minimization to show how cognitive inertia and schema rigidity constrain the brain's capacity to effectively externalize entropy, ultimately contributing to cognitive fatigue.

## 2 Cognitive schemas and automaticity in cognition

This section introduces cognitive schemas as the central organizing structures of the present framework, outlining their role in cognitive stability, automaticity, and prediction.

A schema is an organized mental framework of thoughts or behaviors that structures knowledge about the world. Schemas guide how individuals perceive information from the environment, providing access to past experiences and knowledge needed for specific actions (Neisser, 1976).

In Remembering (1932), Bartlett showed that prior knowledge alters new information, emphasizing that memory retrieval is often reconstructive, linked to mental schemas (Bartlett, 1932). He described schemas as active frameworks derived from prior experiences, functioning as mental templates blending past behaviors and novel actions. Brewer (2000) highlighted Bartlett's recognition of mental representations in navigating complex knowledge.

Cognitive schemas prioritize stability over adaptability, promoting coherence but sometimes hindering flexibility and entropy externalization, concepts will be further examined in relation to cognitive inertia and prediction mechanisms.

### 2.1 Schema activation and the management of errors

In the 1980s, Donald Norman extended the application of cognitive schema, focusing on his work in error taxonomy (Norman, 1981). He examined how the incorrect activation of schemas could lead to unintended actions, resulting in errors. His research identified multiple ways such activation errors could manifest as action-related mistakes. Additionally, Norman and Shallice (1986) developed the Supervisory Attentional System (SAS) model of human performance to explain how everyday activities are influenced by schemas, which they described as behavioral templates activated by environmental cues.

Their model of attention and control suggests that two levels of control exist for well-learned action sequences: deliberate, conscious control and automatic processing. The Supervisory Attentional System (SAS) connects environmental features to a set of schemas. When a schema is activated, it triggers the selection and execution of responses. Repeated practice strengthens the link between environmental features, schemas, and responses, leading to automatic behavior. This framework, referred to as contention scheduling, focuses on prioritizing competing schemas (Cooper and Shallice, 2000). The system resolves conflicts by activating supportive schemas while inhibiting conflicting ones. Although multiple schemas can be activated simultaneously, the target schema is selected based on the strength of its activation (i.e., whether it surpasses a threshold) and the individual's motivations. For novel or complex tasks, an additional control mechanism is required, involving attention-driven processes to adjust activation levels.

This distinction between automatic and controlled action selection also resonates with dual-process accounts. In Kahneman's terms, fast and automatic processing (“System 1”) tends to rely on well-learned, schema-driven routines, whereas controlled processing (“System 2”) supports deliberate override when habitual responses become maladaptive (Kahneman, 2011). Within the present framework, excessive reliance on schema-driven automaticity can therefore be understood as a functional expression of schema rigidity, increasing the cost of control when environmental demands require updating or inhibition.

Classical conditioning provides an early behavioral model of schema formation. Pavlov's experiments with dogs demonstrated how repeated associations between a neutral and a biologically relevant stimulus lead to the establishment of a stable cognitive framework. According to Rescorla (1988), the effectiveness of Pavlov's conditioned reflex depends not merely on the temporal proximity of stimuli but on the predictive value of the conditioned cue. This mechanism parallels the process of schema consolidation, in which repeated exposure fosters the anticipation of outcomes and the automation of cognitive responses.

### 2.2 Predictions and cognitive inertia

As part of the perceptual cycle, Neisser (1976) describes the cyclical interaction between individuals and their environment, highlighting how schemas act as predictive mechanisms that shape perception, guide decision-making, and influence actions. Supported by the principle of Prediction Error Minimization (PEM), as proposed by Hohwy (2016), the brain continuously strives to reduce the discrepancy between predicted sensory input, based on its internal model of the world, and the actual sensory input (Friston and Stephan, 2007). This proactive process involves individuals seeking information to refine their schematic structures, aligning them more effectively with the environment (Hohwy, 2016). While this mechanism supports cognitive stability and coherence, it also reinforces a form of cognitive inertia, wherein established schemas resist change except when a significant environmental stimulus occurs, even in the face of novel or shifting environmental demands (Wei, 2023). Wei (2023) identifies a connection between cognitive schemas and physical mass. Drawing on Newton's first law of motion (Newton, 1687), which states that an object will remain at rest or continue moving uniformly unless acted upon by an external force, Wei parallels this concept to the development of human mental schemas. Environmental stimuli are likened to forces that influence the formation of these schemas, while size or extent of the mental schemas is analogous to mental inertia. Thus, cognitive inertia signifies a person's propensity to continue engaging in repetitive behaviors, which may be linked to specific actions.

Consequently, cognitive schemas tend to preserve existing actions and automatic processes. This view echoes Piaget's theory of cognitive development, in which schemas evolve through processes of assimilation and accommodation (Piaget, 1964). While these mechanisms enable adaptation, they also lead to structural stability once equilibrated. In this framework, cognitive inertia can be interpreted as the persistence of equilibrated schemas that resist modification unless disrupted by significant environmental input. Such rigidity, while promoting coherence, limits flexibility and contributes to the buildup of internal entropy in dynamic contexts.

Such rigidity can limit the system's ability to adapt dynamically to changes, restricting the externalization of entropy by prioritizing internal consistency. Kahneman and Tversky's (1979) research on decision-making further illustrates that human cognition is not always rational, with decisions often reflecting pre-existing schemas rather than active adaptation to new circumstances. This highlights how cognitive inertia may undermine flexibility.

This paper hypothesizes that schema rigidity hinders the brain's ability to externalize entropy in dynamic contexts, limiting behavioral adjustments and increasing cognitive load. Understanding how rigidity impacts adaptability offers pathways for interventions enhancing schema flexibility, reducing internal disorder, and improving performance in complex environments.

## 3 Entropy and free energy principle

To elaborate on the framing outlined in the introduction, this section clarifies that Friston's Free Energy Principle, formulated in information-theoretic terms, provides the central explanatory model, while thermodynamic principles are considered only at the level of the material organization of biological systems.

Biological systems, such as the brain, rely on the continuous regulation of energetic and entropic exchanges with their environment to maintain internal stability (Friston, 2013; Rabeyron, 2021). This dynamic process ensures that order is sustained despite constant external demands. Drawing on principles from physics and biology, particularly the concepts of entropy and free energy, we can better understand how living systems sustain internal order through interaction with their surroundings.

In what follows, I consider two frameworks in parallel. Classical thermodynamics, particularly the notion of open systems that exchange mass (and, by implication, energy and entropy) with their environment, describes the physical constraints sustaining biological organization. By contrast, Friston's Free Energy Principle relies on information-theoretic notions of entropy and free energy to account for cognitive inference and prediction.

### 3.1 Entropy and free energy in classical thermodynamics

In classical thermodynamics, entropy characterizes the irreversibility of macroscopic processes and determines the direction of spontaneous evolution. Entropy can be defined phenomenologically, following Clausius (1865), or statistically, following Boltzmann (1877), as a measure of the number of microscopic configurations compatible with a given macroscopic state: the larger this number, the more probable and closer to equilibrium the state. Free energy, formulated as either Helmholtz or Gibbs free energy depending on the constraints, combines energy and entropy into a single quantity that determines equilibrium and spontaneous change in physical systems. It represents the portion of a system's energy available for macroscopic work once entropic constraints are taken into account. Thus, entropy links microscopic dynamics to macroscopic irreversibility, while free energy governs the direction and limits of physical transformations (Lebowitz, 1993; Gibbs, 1902).

A classical illustration of entropy increase is the spontaneous diffusion of a gas initially confined to a restricted volume. Once the constraint is removed, the gas expands to occupy the available space, reaching a macroscopic state compatible with a vastly larger number of microscopic configurations. The reverse process does not occur spontaneously, exemplifying the probabilistic nature of the second law of thermodynamics.

This example illustrates the necessity of constraints and energy input to establish an organized state. In biological systems, maintaining organization also depends on continuous exchange with the environment and entropy export.

The brain is a thermodynamically open system in the classical sense, exchanging mass—such as O_2_, CO_2_, glucose, and other metabolites—with its environment. Through these exchanges, biological organization is locally maintained while entropy is exported to the surroundings. The relevance of this material description for cognitive processes will be addressed in the following section.

By transferring or “externalizing” entropy to their surroundings, they expel disorder and maintain internal order, a process physicist Erwin (Schrödinger 1944) described as “negative entropy” or “negentropy.” Schrödinger highlighted that living systems sustain themselves far from thermodynamic equilibrium by extracting energy and order from their environment—for instance, through the consumption of food or sunlight—and by expelling entropy back into the surroundings. This ongoing exchange allows biological systems to resist the natural drift toward disorder predicted by the second law of thermodynamics. Without this continuous input of low entropy energy, organisms would inevitably succumb to increasing internal entropy and disorganization.

This natural tendency toward entropy illustrates why order and stability cannot arise spontaneously without an external energy input.

### 3.2 Informational entropy and free energy in cognition

Building on this thermodynamic foundation, Friston extends the notion of entropy regulation into the informational domain. In cognitive systems, “entropy” no longer quantifies physical disorder but uncertainty—the unpredictability of internal states relative to environmental input. In the context of the Friston's Free Energy Principle, entropy refers to the expected surprise—the average uncertainty about the organism's sensory and internal states.

Within Friston's framework, informational entropy expresses the system's uncertainty about the causes of its sensory inputs. High entropy corresponds to a state of unpredictability, where the organism cannot reliably infer or anticipate environmental changes. Conversely, low entropy reflects stable and predictable interactions between the organism and its environment, allowing it to preserve physiological and cognitive equilibrium. Thus, minimizing informational entropy is equivalent to maintaining a state of viability—a condition of adaptive stability that underlies survival (Friston, 2010, 2013).

It is not a thermodynamic quantity but an information-theoretic measure expressing how predictable or stable the system's states are relative to its environment (Friston, 2010).

At a higher level, this principle of entropy regulation can be extended to cognitive processes. The brain, as an open biological system, actively reduces informational uncertainty (entropy) by continuously predicting and adapting to changes in its environment (Friston, 2009). This adaptive process, formalized in the free energy principle, ensures that organisms maintain internal order and stability.

When this regulatory process falters, it may give rise to phenomena such as cognitive fatigue, which can be understood as a breakdown in the balance between order and disorder. This imbalance, I argue, stems from cognitive rigidity and the inertia of established schemas, which constrain adaptability and impair performance. To better understand this dynamic, it is useful to consider how Friston formalized this regulatory process within his Free Energy Principle.

Friston (2013) further formalized this principle mathematically, demonstrating how living systems regulate entropy through their internal structures and states. A failure in this regulatory activity leads to an increase in free energy within the system, disrupting homeostasis. The free energy principle posits that organisms minimize free energy by reducing prediction errors, enabling them to better anticipate and adapt to environmental changes (Friston, 2009; Kaplan and Friston, 2018).

Central to this framework is the concept of “organizational closure,” described by Maturana and Varela (1987), where biological systems create boundaries (e.g., a cell membrane) to separate internal states from external conditions. This boundary facilitates homeostasis and regulates interactions with the environment. Friston introduced the concept of the “Markov blanket,” a mathematical construct that formalizes these boundaries by defining the exchange between internal and external states. The Markov blanket acts as a filter, enabling the system to infer external causes while preserving internal stability (Cieri and Esposito, 2019). While the Markov blanket defines the fundamental boundary for all living systems, its relevance is especially evident in the brain, where it structures the dynamic interplay between internal models and external stimuli.

In humans, this principle extends to the brain's generative model, a hierarchical structure that simulates the environment to make predictions and improve adaptive capacities (Friston, 2009). At a lower level, sensory inputs may inform the brain about immediate changes in the environment, while higher levels integrate this information into more abstract models, such as goals or contextual understanding.

At each hierarchical level, the Markov blanket serves as a boundary separating internal from external stimuli, enabling the reduction of entropy and free energy. Each level processes and regulates prediction errors from lower levels, reducing both entropy and free energy (Connolly and van Deventer, 2017). When this hierarchical regulation fails, due to rigid generative models or inefficient prediction updates, it increases free energy and reduces performance, contributing to phenomena like cognitive fatigue. The hierarchical, recursive nature of the generative model illustrates how the Free Energy Principle applies across different scales, from biological mechanisms to psychological processes. This reflects the brain's ability to minimize prediction errors while maintaining stable internal organization, regardless of the level of analysis.

When entropy externalization diminishes, the system struggles to maintain internal organization, leading to performance declines. This aligns with Kaplan and Friston's (2018) perspective that planning depends on the efficient regulation of entropy and prediction errors to respond to environmental uncertainties. Disruptions create inefficiencies in managing demands, reducing the system's capacity for dynamic reorganization.

Building on this framework, cognitive schemas play a key role in structuring thought and behavior. These schemas enable efficient information processing by reducing uncertainty and becoming automatic over time. While supporting stability, their inherent rigidity can hinder updates to internal models, a process essential for adapting to environmental changes.

### 3.3 Entropy in the proposed framework

In the present theoretical framework, entropy refers to informational uncertainty. This definition, consistent with the information-theoretic usage proposed by Friston (2010, 2013), is applied here to cognitive dynamics. Entropy quantifies the degree of unpredictability or instability within internal cognitive models as they interact with environmental demands.

Within this framework, cognitive schemas operate as organizing structures that minimize entropy by integrating past experiences and guiding perception, prediction, and behavior. When schemas are flexible, they facilitate the continuous updating of internal models, aligning predictions with sensory input and sustaining cognitive equilibrium. In contrast, schema rigidity constrains this adaptive process: when established cognitive patterns resist modification, prediction errors accumulate, internal uncertainty increases, and informational entropy rises. The growing divergence between internal representations and environmental information disrupts the system's capacity to maintain coherence and contributes to cognitive fatigue. In this sense, cognitive fatigue can be interpreted as the experiential manifestation of an entropic imbalance—a failure of the cognitive system to externalize uncertainty and restore internal order.

Externalizing entropy refers to reducing informational uncertainty through brain–environment coupling via belief updating (and, on longer timescales, learning/schema revision) and via adaptive action that changes sensory sampling to obtain uncertainty-reducing evidence. Schema rigidity limits these mechanisms by reducing the capacity to revise entrenched priors and to flexibly adjust information sampling.

To situate this entropy-based account, it is useful to briefly note key limitations of existing approaches in the cognitive-fatigue literature.

Early theories, including resource depletion accounts, attribute cognitive fatigue to reduced internal resources (e.g., glucose; Baumeister et al., 1998, 2018, 2007; Caggiano and Parasuraman, 2004; Matthews, 2011; Muraven et al., 2006). The empirical association between glucose fluctuations and cognitive performance is not consistent, which complicates strong depletion interpretations (Beedie and Lane, 2012; Kurzban, 2010; Molden et al., 2012; Sanders et al., 2012). Dual-regulation accounts also remain unclear about the definition and implementation of facilitation and inhibition mechanisms (Ishii et al., 2014).

Motivational models, including Hockey's (2011) framework, suggest that fatigue reflects conflicts between sustaining task focus and reallocating attention toward alternative activities. Related perspectives propose that fatigue emerges from changes in effort allocation guided by cost–benefit considerations (Boksem and Tops, 2008; Inzlicht et al., 2014; Inzlicht and Schmeichel, 2012; Kurzban et al., 2013; Milyavskaya and Inzlicht, 2017). Such reallocations may reduce commitment to long-term goals while increasing sensitivity to immediate rewards (Boat and Cooper, 2019). Recent meta-analytic evidence, though, provides limited support for the claim that cognitive fatigue reliably produces motivational shifts across paradigms (Hunte et al., 2024), underscoring the need for further empirical clarification.

A neurochemical perspective highlights the role of neurotransmitters, such as dopamine and adenosine, in sustaining cognitive performance during effortful tasks (Arenales Arauz et al., 2024). Adenosine buildup in the anterior cingulate cortex reduces neuronal efficiency, reflecting the metabolic cost of sustained effort. Overall, these approaches remain limited in that they do not explicitly model the brain's dynamic interaction with its environment.

## 4 How cognitive schema rigidity impairs entropy externalization

Building on the previous discussion of informational entropy, this section examines how the rigidity of cognitive schemas limits the brain's ability to regulate and externalize entropy. Once cognitive schemas become excessively stable, their function shifts from reducing uncertainty to constraining adaptability. This rigidity impedes the system's capacity to adjust internal models to environmental demands, increasing informational disorder and contributing to cognitive fatigue.

Within the context of cognitive schemas and their automatic properties, mental inertia can be characterized by its capacity to facilitate learning through repeated actions, its activation by content-specific or script-based processes, and its role in promoting the emergence of automatic behaviors (Haggar et al., 2019). Cognitive rigidity can be likened to physical inertia, as described by Newton's first law (Newton, 1687). This law states that an object remains at rest or in uniform motion unless acted upon by an external force. Similarly, cognitive schemas, shaped by environmental stimuli, exhibit stability and resistance to change. External stimuli must exceed the threshold of mental inertia to induce adjustments in these mental structures and corresponding behaviors. According to Newton's first law, when external stimuli are sufficiently powerful to overcome mental inertia, they can prompt changes in mental structures, resulting in behavioral adaptations. Conversely, if these stimuli fail to surpass the threshold of mental inertia, no significant alterations in mental patterns or behaviors will occur (Wei, 2023). In such a scenario, the individual remains trapped in a closed, non-stimulating system where dynamic interactions with the environment are minimal. This lack of external engagement prevents the necessary exchange of information and adaptation, causing the system to drift toward entropy. A similar phenomenon was observed in Kamin's (1969) blocking experiment, where pre-existing associations between a stimulus and a response prevented the learning of new associations with a secondary stimulus. This suggests that rigid cognitive schemas might block the integration of new environmental inputs, reinforcing internal rigidity.

In this context, it is reasonable to hypothesize that such rigidity and the absence of dynamic interaction with the environment could reduce the ability to externalize entropy, which may in turn increase cognitive load and ultimately affect performance. If schemas are conceptualized as stable cognitive structures comparable to traits (Young, 1990), their stability and persistence could be assessed and measured.

Extending this analogy further, Ernst Mach's proposition that a particle's inertial mass is influenced by surrounding masses (Mach, 1872) and Volkmar Putz's assertion that inertia is entirely dictated by external gravitational forces (Putz, 2019) suggest parallels in psychology. Cognitive schemas can similarly be understood as shaped by external environmental stimuli. The magnitude of mental inertia reflects the degree of resistance to external forces, highlighting the system's reliance on environmental inputs for adjustment (Wei, 2023).

The Psychological Momentum Theory (PMT) suggests that an individual's response rate is proportional to the intensity of external stimuli or interventions. This framework uses response rate, or velocity, as a proxy for mass or inertia (Deemer et al., 2021). However, distinguishing between high velocity (active engagement) and high mental inertia (resistance to change) remains challenging. Momentum, defined as the product of mass and velocity, underscores the importance of both factors in behavioral dynamics (Markman and Guenther, 2007).

Most studies measure cognitive fatigue through a decline in performance, such as slower reaction times or higher error rates. Yet, these approaches do not address the underlying role of mental inertia. High mental inertia, characterized by the persistence of rigid schemas, could make the externalization of entropy particularly difficult. This resistance to schema adjustment forces the brain to manage internal disorder, contributing to an accumulation of cognitive load. For instance, individuals in a state of high velocity may demonstrate rapid engagement with tasks but lack the rigidity that characterizes high mental inertia. Conversely, high mental inertia reflects sustained repetitive behaviors with significant resistance to change. Understanding this distinction is crucial for assessing the impact of cognitive inertia on behavioral adaptation and fatigue.

Rigid cognitive schemas limit the system's ability to externalize entropy, forcing the brain to manage internal disorder. This accumulation of cognitive load arises when environmental stimuli fail to prompt schema adjustments, trapping the individual in repetitive and ineffective behavioral cycles. While this stability may be beneficial in predictable environments, it becomes a liability in dynamic or unpredictable contexts, where adaptability is critical.

This perspective aligns with my hypothesis that high mental inertia contributes to cognitive fatigue by restricting flexibility and increasing internal entropy. Measuring mental inertia in terms of schema stability and resistance to external stimuli provides a pathway for future research to explore interventions aimed at enhancing cognitive flexibility. By facilitating entropy externalization, such interventions could reduce cognitive load and improve performance in demanding environments.

## 5 How rigid cognitive schemas contribute to cognitive fatigue accumulation

**Figure 2:**
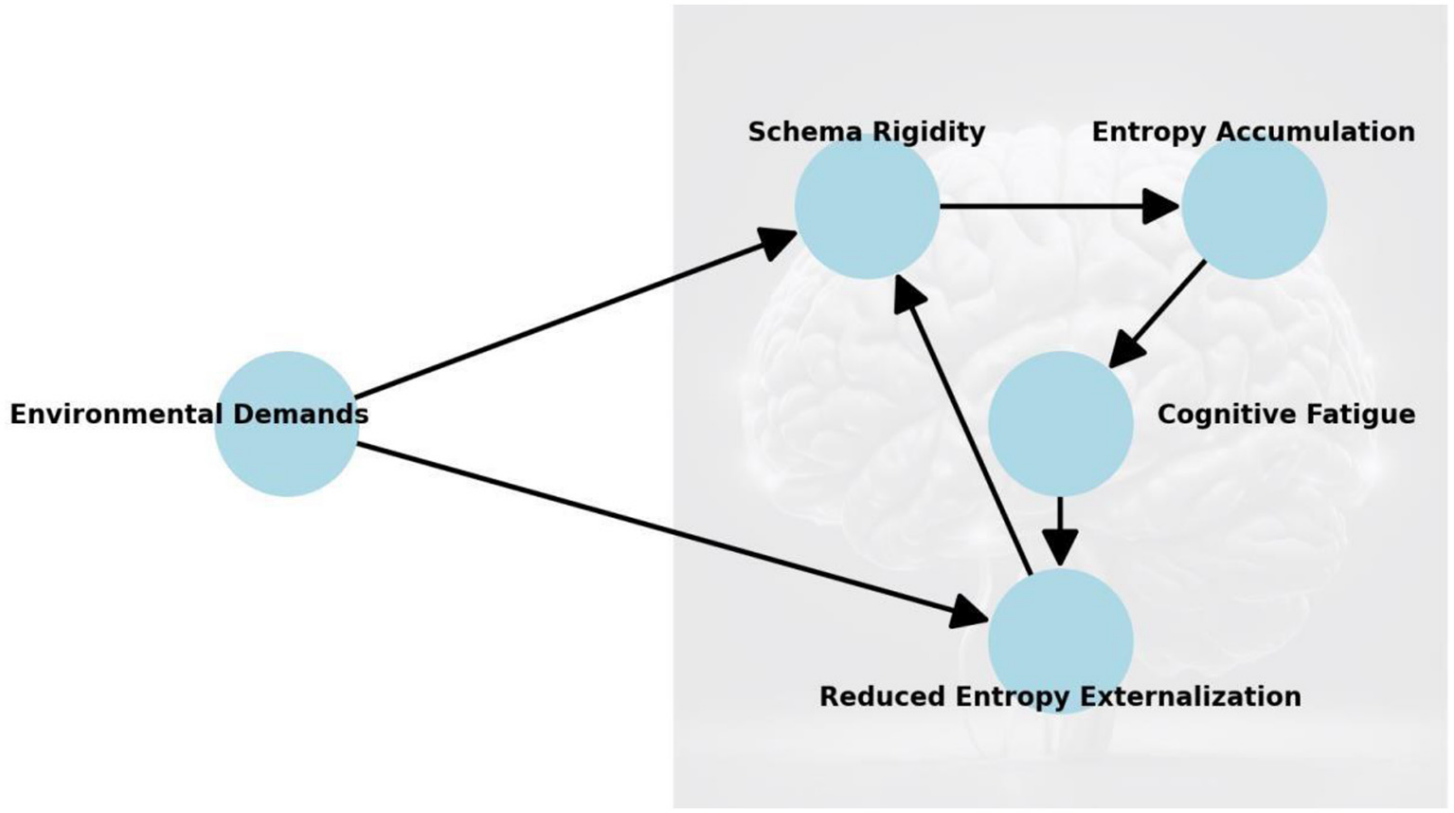
*Cognitive fatigue* as a phenomenon emerging from the interaction between cognitive *schema rigidity, increasing entropy*, and imbalances between internal processes and *external environmental demands*. Starting with *Environmental Demands*, which represent external influences, these demands affect both *Schema Rigidity* and the brain's ability to *Externalize Entropy*. The rigidity of cognitive schemas leads to *Entropy Accumulation*, which contributes to *Cognitive Fatigue*. Fatigue, in turn, *reduces entropy externalization*, creating a feedback loop that reinforces *schema rigidity*. The arrows represent causal relationships, showing how each factor influences the next, forming a self-reinforcing cycle that exacerbates *cognitive fatigue*.

Building upon the elements and dynamics of cognitive schemas defined earlier, this section examines how their rigidity disrupts entropy regulation and leads to cognitive fatigue. The perspective of this paper builds on the theoretical framework above to investigate the interplay between cognitive schema rigidity, entropy accumulation, and cognitive fatigue (Figure 2).

Cognitive fatigue arises not only from task complexity or resource depletion but also from the inability to adapt dynamically to changing demands. At its core, it stems from schema rigidity, which prioritizes stability over flexibility. While beneficial in predictable contexts, this rigidity becomes maladaptive in dynamic environments, amplifying prediction errors and internal entropy, ultimately increasing cognitive load.

Rigid schemas serve as stable mental frameworks that resist modification, even when external cues demand change. This is consistent with dual-process accounts in which automatic, schema-driven responses can dominate when control is costly, increasing reliance on rigid routines under changing demands (Kahneman, 2011). In the present framework, the key consequence is that rigidity constrains model updating, allowing prediction errors to persist and informational entropy to accumulate, thereby amplifying cognitive load and fatigue. The brain's failure to externalize entropy effectively—by adapting internal states to meet environmental demands—further amplifies this burden (Hesp et al., 2021). According to Friston's Free Energy Principle, biological systems aim to minimize free energy (encompassing prediction errors and entropy) to maintain stability (Friston, 2009). Rigid schemas restrict this process, forcing the brain to manage internal entropy, increasing cognitive strain and fatigue.

For example, in the Stroop task (Stroop, 1935), entrenched schemas such as the automaticity of reading hinder adaptation to incongruent attributes of the same stimulus (word meaning vs. ink color). This rigidity prevents the updating of internal models, increasing cognitive effort to override prior beliefs (e.g., “words should be read”) with sensory evidence, thereby reducing adaptability and amplifying fatigue (Friston et al., 2017; Kool and Botvinick, 2014). Over time, this unresolved entropy accumulates, intensifying cognitive load.

A Stroop-like real-world analog is mode-based automation in aviation: pilots' well-learned *input*→*outcome* expectations are typically reliable, yet can be intermittently violated when automation changes modes—sometimes with subtle or weakly signaled state transitions. Under such conditions, the same action can yield an unexpected system response, increasing the need for inhibition, mode diagnosis, and rapid re-alignment between the pilot's internal model and the automation's actual state. Recurrent schema–reality conflicts of this kind can accumulate cognitive load.

Within the framework of active inference, rigid schemas act as dominant prior beliefs that overshadow inference processes, making it energetically costly to revise predictions when faced with novel or conflicting stimuli (Friston et al., 2017). This rigidity limits adaptability and traps the brain in maladaptive cycles of cognitive effort, increasing internal entropy and fatigue.

While traditional models of cognitive fatigue often focus on task difficulty or resource depletion, this perspective highlights schema rigidity as a key factor that can impair performance and adaptability. By failing to update schemas, the system becomes increasingly burdened by unresolved internal disorder. Addressing these dynamics calls for targeted interventions that enhance schema flexibility, reduce entropy, and improve adaptability in complex environments.

## 6 Discussion

This article introduces a novel argument for understanding cognitive fatigue, grounded in Friston's Free Energy Principle and the second law of thermodynamics. Unlike traditional models that emphasize resource depletion or motivational shifts, this framework views cognitive fatigue as a consequence of the brain's difficulty in dynamically adapting to environmental demands. Cognitive schemas serve as stabilizing structures that reduce uncertainty by organizing information and guiding behavior. When these schemas become excessively rigid, they limit the brain's capacity to adapt to novel or changing contexts, leading to an accumulation of unresolved internal entropy and increased cognitive load.

One of the most significant contributions of this article is its emphasis on the critical role of brain-environment interactions—a perspective that remains largely untested in cognitive fatigue research despite its growing relevance in contemporary theoretical advancements. The Free Energy Principle (Friston, 2013), which is increasingly cited in cognitive neuroscience, highlights the importance of dynamic exchanges between the brain and its surroundings to maintain stability and reduce entropy. Despite this, existing models of cognitive fatigue have yet to incorporate this critical interaction, focusing instead on internal processes like resource depletion. This omission represents a significant gap in the literature. By linking cognitive fatigue to the brain's inability to externalize entropy due to schema rigidity, this framework addresses this gap and offers a novel direction for future research.

Traditional models of cognitive fatigue, such as the resource depletion model (Baumeister et al., 1998, 2018), suggest that cognitive performance declines as internal resources are consumed over time. This assumption has been challenged by evidence questioning a direct link between glucose levels and performance (Kurzban, 2010; Molden et al., 2012). Motivational models (Hockey, 2011) propose that fatigue reflects a conflict between maintaining task focus and shifting attention to alternative activities. While these models have provided valuable insights, they largely neglect the dynamic interaction between cognitive processes and environmental demands. The proposed framework integrates principles from physics, biology, and cognitive science to offer a more holistic perspective. By viewing the brain as a thermodynamically open system that continuously exchanges entropy with its environment, this model emphasizes the importance of schema flexibility in managing cognitive load. It builds on the idea that cognitive fatigue arises not solely from internal depletion but from a failure to adapt internal models to changing external conditions.

The practical implications of this framework suggest that interventions aimed at reducing cognitive fatigue should focus on enhancing schema flexibility rather than solely on resource replenishment. Cognitive training programs could incorporate exercises that promote adaptability by encouraging individuals to engage with novel or unpredictable scenarios. Work environments that promote dynamic interactions and reduce repetitive, monotonous tasks could help mitigate cognitive fatigue by preventing the entrenchment of rigid schemas.

While this article presents a novel perspective on cognitive fatigue, it remains a theoretical framework that requires empirical validation. Future research should investigate the relationship between schema rigidity and cognitive fatigue using tasks that require dynamic adaptation, such as virtual navigation tasks in unpredictable environments or simulations where participants must continuously adjust to changing conditions. Simulations based on the Free Energy Principle could model how entropy externalization influences cognitive performance over time. Developing interventions to enhance schema flexibility could reduce cognitive load and improve performance in demanding environments. By integrating insights from multiple disciplines, future studies could refine this framework and contribute to a more comprehensive understanding of cognitive fatigue as a systemic phenomenon.

## 7 Conclusions and future directions

This theory advances a schema-rigidity/entropy-regulation account of cognitive fatigue grounded in predictive processing and Friston's Free Energy Principle. Cognitive schemas support efficient, stable perception–action by constraining inference and guiding attention and behavior. When cognitive inertia renders schemas excessively rigid, adaptive model updating is limited under changing environmental demands. As a result, prediction errors persist, informational entropy (uncertainty) accumulates, and fatigue emerges as the system increasingly struggles to restore coherence through effective brain–environment exchange and entropy externalization. These mechanisms have practical implications in several applied settings.

For example, in aviation, operational environments are increasingly shaped by automation, including the growing integration of AI. Under this account, rigid schemas make operators especially vulnerable to cognitive fatigue when automation behaves in ways that violate entrenched expectations (e.g., unexpected actions, mode transitions, or abrupt context shifts). Accordingly, before deploying or scaling automation, evaluation should prioritize identifying situations most likely to amplify schema–reality conflict and ensuring that the system supports rapid re-alignment between the operator's model and the automation's actual state. Future studies could evaluate whether interface designs that reduce hidden states and increase transparency of mode transitions measurably decrease prediction-error persistence, informational entropy, and fatigue during prolonged supervisory control.

## Data Availability

The original contributions presented in the study are included in the article/supplementary material, further inquiries can be directed to the corresponding author.
